# The simulation of meiosis in diploid and tetraploid organisms using various genetic models

**DOI:** 10.1186/1471-2105-13-248

**Published:** 2012-09-26

**Authors:** Roeland E Voorrips, Chris A Maliepaard

**Affiliations:** 1 Department of Plant Breeding, Wageningen University and Research Center, P.O. Box 16, 6700 AA, Wageningen, the Netherlands

**Keywords:** Population genetic simulation software, Meiosis, Chiasma interference, Tetrasomic inheritance, Bivalents, Quadrivalents, Double reduction

## Abstract

**Background:**

While the genetics of diploid inheritance are well studied and software for linkage mapping, haplotyping and QTL analysis are available, for tetraploids the available tools are limited. In order to develop such tools it would be helpful if simulated populations based on a variety of models of the tetraploid meiosis would be available.

**Results:**

Here we present PedigreeSim, a software package that simulates meiosis in both diploid and tetraploid species and uses this to simulate pedigrees and cross populations. For tetraploids a variety of models can be used, including both bivalent and quadrivalent formation, varying degrees of preferential pairing of hom(oe)ologous chromosomes, different quadrivalent configurations and more. Simulation of quadrivalent meiosis results as expected in double reduction and recombination between more than two hom(oe)ologous chromosomes. The results are shown to match theoretical predictions.

**Conclusions:**

This is the first simulation software that implements all features of meiosis in tetraploids. It allows to generate data for tetraploid and diploid populations, and to investigate different models of tetraploid meiosis. The software and manual are available from http://www.plantbreeding.nl/UK/software_pedigreeSim.html and as Additional files 1, 2, 3 and 4 with this publication.

## Background

Most plant and animal species are diploid: individuals have two homologous sets of chromosomes, one set originating from each of the two gametes from which they were formed. Since the work of Mendel [1] on peas, our understanding of the genetics in diploid species has advanced enormously, and nowadays many statistical tools are available for the analysis of the inheritance of molecular markers and qualitative and quantitative traits in diploids.

However many organisms exist in which some or all chromosomes are present in four copies: partial or complete tetraploids. Some important crop species belong to this group, including potato, rose, leek, cotton and alfalfa. Other ploidy levels also occur but are not the subject of this paper. In a tetraploid the four copies of a given chromosome may be completely homologous or there may be two slightly different types (homoeologues) present that pair more easily with their own type than with the other. The degree of preferential pairing may vary from 0% (fully random pairing, as in autotetraploids where the four copies of a chromosome are completely homologous) to 100% (the homoeologues pair exclusively with their own type, as in allotetraploids). Intermediate forms with partially preferential pairing may also occur [2] but are probably not stable [3,4].

**Figure 1:**
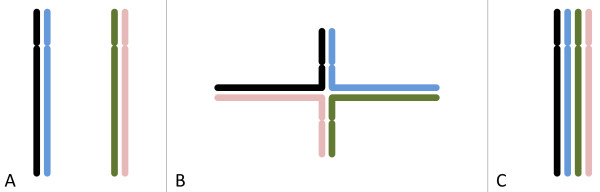
**Three metaphase configurations of one set of homologous chromosomes in tetraploid meiosis. A**. two bivalents; **B**. “cross-type” quadrivalent; **C**. “parallel” quadrivalent. In a cross-type quadrivalent two branches contain the tops of two chromosomes, and two branches the bottoms of two chromosomes; the location of the chromosome exchange point (the position where the branches meet) may vary between meioses.

In diploids, the two homologous chromosomes pair up during meiosis to form a bivalent. In the bivalent crossing-over and recombination occur, and after the first meiotic division the two centromeres, each with two chromatids, are separated. During the second meiotic division the two centromeres split and the four halves, each with one chromatid, end up in four gametes. In tetraploids two configurations occur predominantly. In one configuration the four hom(oe)ologous chromosomes form two separate bivalents, each of which contributes one chromatid to each gamete as in the case of diploids (Figure 1A). Alternatively the four chromosomes can form one quadrivalent in which recombination takes place between the eight chromatids. In the first meiotic division the four centromeres are separated into two pairs. In the second meiotic division the two centromeres at each pole split, and one half of each ends up in each gamete (Figure 1B, C). Both in the case of two bivalents and in the case of a quadrivalent each gamete contains two chromatids, with the centromeres originating from two different parental chromosomes. Some other meiotic configurations occur as well, but all of those involve univalents (unpaired chromosomes) and have a high probability of producing unbalanced gametes; they are not considered here.

The genetics of tetraploids are more complicated than those of diploids because in an individual more than two alleles can be present at the same locus, the dosage of an allele can vary from 0 to 4 copies, there are more phase and recombination possibilities and preferential pairing may occur to varying degrees. An additional meiotic complication in tetraploids is the occurrence of double reduction: the situation where one gamete receives two copies of part of the same parental hom(oe)olog (see next section). In comparison with diploids, the software tools for genetic analyses in tetraploids are less developed. For assessing the allele dosage in SNP assays several software packages were published recently: beadarrayMSV [5], fitTetra [6] and SuperMASSA [7]. In diploids much software is available for linkage analysis and QTL mapping; a few widely used packages are MAPMAKER [8], JoinMap [9] and MapQTL [10]. For the generation of tetraploid linkage maps and haplotype phasing the TetraploidMap software [11] was developed. However, compared with the packages for diploids mentioned above the functionality and power are less, there are severe restrictions on the number of markers that can be handled, much manual interaction and visual inspection is needed and not all combinations of parental marker genotypes can be handled. This makes the use of the software problematic for situations with large numbers of markers, as in the case of SNPs.

In order to test algorithms and develop software for genetic analysis in tetraploids it would be useful to simulate the genetic and phenotypic composition of tetraploid populations. However, as far as we are aware the only publicly available simulation software for tetraploids is Polylink [12], and this is limited in that it does not simulate gamete formation through quadrivalents. Here we discuss the various models of tetraploid meiosis that have been described in literature, and we present a new software package PedigreeSim that simulates the process of gamete formation in diploids and tetraploids and uses this to generate simulated cross progenies and pedigrees. To our knowledge this is the first published simulation package that includes simulation of gamete formation in quadrivalents and hence is able to simulate double reduction.

## Implementation

### Simulation of meiosis

Our simulation of meiosis follows the steps that occur in nature. First, in a tetraploid it must be decided for each chromosome whether the four homologs will form two bivalents or one quadrivalent; in diploids the two homologs always form a bivalent (in our simulations we ignore the possibility of other configurations, which always involve univalents or unpaired chromosomes and have a high chance of producing unbalanced gametes and are less likely to result in viable progeny). Second, in the bivalents or quadrivalents crossover events are generated (hereafter for brevity we refer to these as chiasmata), resulting in recombined chromatids that are mosaics of the parental chromosomes. Third, the centromeres and attached chromatids are separated as in the first meiotic division. Finally the centromeres are split and distributed with the attached chromatid to the gametes as in the second meiotic division. These steps are taken to be independent between the different sets of hom(oe)ologous chromosomes in the same meiosis. Next we consider these steps in more detail.

#### Step 1: bivalents or quadrivalent?

In diploids, each pair of homologous chromosomes forms a bivalent; for the simulation of meiotic recombination and segregation it is not relevant how these bivalents are formed. For tetraploids, a simple but in many species acceptable model for the association of hom(oe)ologous chromosomes is that pairing starts independently at the telomeres and proceeds inwards [13,14]. If all pairwise combinations are equally likely, then in 1/3 of the cases two bivalents are formed (Figure 1A) while 2/3 of the cases result in a quadrivalent with one point of chromosome exchange ( [14]; Figure 1B). In [15] numerous tetraploid plant species are cited where this ratio is actually observed. If preferential pairing of the telomeres occurs the ratio of quadrivalent to bivalents is decreased, with the exclusive formation of bivalents in the extreme case of 100% preferential pairing.

Apart from this “natural” approach where the ratio of bivalents to quadrivalents is determined by the degree of preferential pairing, our software also offers the possibility to specify this ratio explicitly per chromosome. In that case we first determine which configuration to generate, and within the constraints of that configuration we perform the pairing (meaning, for example, that if two bivalents are to be generated the pairing procedure is performed only at one of the telomeres with the opposite telomeres following the first telomere).

The quadrivalents generated by these approaches have four branches, in two of which the “top” ends of two chromosomes are paired, while in the other two branches two “bottom” ends are paired; each branch has a different chromosome combination (Figure 1B). The chromosome exchange points occur at different positions, so that the lengths of the “top” and “bottom” branches vary between quadrivalents. We shall use the term “cross-type quadrivalent” to designate this configuration. In each branch recombination can occur only between two chromosomes. Another configuration can be imagined where all four chromosomes are arranged in parallel, so that at any point recombination can involve any pair of chromosomes (Figure 1C). Such a model is discussed in [16]; however this type of quadrivalent pairing does not seem to occur often [17]. Still our software allows to specify the ratio of “cross-type” versus “parallel” quadrivalents.

In contrast to a pair of bivalents, a quadrivalent can give rise to recombined chromatids consisting of segments from three or, in the case of parallel quadrivalents, all four parental chromosomes. Also double reduction can occur in quadrivalents: the situation where a part of the two hom(oe)ologous chromatids in one gamete is derived from the same parental chromosome; a parent with four different alleles at a certain locus can thus produce gametes that are homozygous for one of these alleles. A clear description of double reduction is given in [18]. These situations are reproduced in our simulations as discussed in the Results and Discussion section.

#### Step 2: generation of chiasmata

A chiasma is specified by the two involved chromatids and the crossover position. In the following discussion we take a Morgan to mean the length of a chromosome segment on which an average of one chiasma per chromatid occurs per meiosis. The Morgan was defined originally in terms of recombination frequencies in diploids [19], but as the amount of recombination corresponding to a certain number of chiasmata in a tetraploid varies according to the meiotic configurations we use the definition mentioned above; for bivalents the definitions are equivalent. According to this definition, in a bivalent on average two chiasmata occur per Morgan. The same chiasma frequency is used in each branch of a cross-type quadrivalent, where like in a bivalent at any point two chromosomes are paired. In a parallel quadrivalent on average four chiasmata per Morgan are generated to obtain the same frequency of recombination points per chromatid. In our approach no chromatid interference is modelled: each chiasma can involve any combination of paired non-sister chromatids with equal probability, independent of other chiasmata.

In contrast, chiasma interference can be modelled with our software. In the absence of chiasma interference the distance between two successive chiasmata follows an exponential distribution, with a mean of 0.5 Morgan (bivalent and cross-type quadrivalent) or 0.25 Morgan (parallel quadrivalent). In this case, in diploids the Haldane map function [19] is applicable. When chiasma interference is modelled it follows the relation of Kosambi’s map function [20]. This is achieved by using a gamma distribution for the distance between successive chiasmata, with shape parameter 2.63 (empirically determined by unpublished simulation experiments) and a scale parameter of 2.63/0.5 (bivalents, cross-type quadrivalents) or 2.63/0.25 (parallel quadrivalents) to obtain a mean distance between chiasmata of 0.5 Morgan or 0.25 Morgan respectively.This approach is different from that in other simulation software, where a chromosome is a priori subdivided into small segments, and where for each segment a chiasma is generated or not, with a probability depending on the segment length and, if chiasma interference is modelled, on the presence of chiasmata in the preceding segments (e.g. [21]). The advantages of our approach are that a chiasma has an exact location so that even high-density marker data can be modelled without affecting the chiasma-generating process, and that the same recombinant chromosomes can be re-used with other marker maps. The Plabsoft simulation package [22] like our software assigns exact locations to chiasmata, but it does not handle chiasma interference and is not able to simulate tetrasomic inheritance.

Chiasmata are generated starting from one telomere, proceeding to the other. In the case without interference the position of the first chiasma is determined by taking a random sample from the exponential distribution and adding that to the chromosome start position. However, in the case of chiasma interference that method cannot be applied, as the position of the next chiasma is affected by the position of the previous chiasma. Therefore we let the process of chiasmata generation “burn in”, by generating a first virtual chiasma position from an exponential distribution starting several Morgan before the start of the chromosome, and succeeding chiasmata by sampling distances from a gamma distribution until the first chiasma is generated beyond the chromosome start.

#### Step 3: First meiotic division

In a diploid the two homologous centromeres are separated from each other and move with the attached chromatids to opposite poles of the cell. In a tetraploid where the four homologous chromosomes have formed two bivalents, each bivalent is separated as in a diploid situation, i.e. from each bivalent one centromere moves to either pole of the cell. In a cross-type or parallel quadrivalent two of the four homologous centromeres move to each pole. The formation of the pairs is apparently random, with each pairing having equal probability [18]; J. Sybenga, pers. comm. This is the default model in PedigreeSim, but for cross-type quadrivalents we offer also a different model in which two paired centromeres always end up at opposite poles in the first meiotic division.

#### Step 4: Second meiotic division, formation of gametes

At the end of the first meiotic division half of the centromeres have ended up at each pole of the cell. Each centromere carries two chromatids, which due to recombination may not be identical anymore. In the second meiotic division each centromere is split in half, and the two halves, each with one chromatid are separated to end up in two different gametes. The separation of the two chromatids of a chromosome is independent of that in any hom(oe)ologous chromosomes and also of the segregation of the non-hom(oe)ologous chromosomes.

### Generation of genotypes

The process described in the previous section generates gametes in which each chromosome is a product of recombination between the homologous chromosomes in the parent. Two further steps are required to derive observable genotypes (e.g. marker genotypes) from these parental recombination products.

The first step is to express each gamete as a mosaic of founder haplotypes. The simulated population consists of founder individuals and offspring. Each homolog of a founder individual has the same “founder allele ID” over its full length. For example, in a diploid population the first founder has two homologs of every chromosome, of which one has the founder allele ID 0 and the other has founder allele ID 1 at every position; the second founder individual has founder alleles 2 and 3, and so on. A chromosome in a gamete or an offspring individual consists of one or more segments of founder chromosomes. For example, a gamete of the second founder individual might have a chromosome consisting of three segments, the first with founder allele 2, the second with founder allele 3 and the third again founder allele 2 (reflecting two recombination events). In this way every chromosome in the population can be described as a sequence of segments, each segment characterized by a founder allele and a start position.

The second step is to define a genetic map for the population and to assign an observable allele to every founder chromosome at every locus on the map. These observable alleles can be anything, including a letter representing a nucleotide at a SNP position, a fragment length for an SSR marker, or a dominant or recessive (A/a) allele of a gene. Given the map and the alleles corresponding to each founder allele, for each individual the ordered observable genotype at every locus is calculated, and also the allele dosage of one of the alleles. This procedure allows all possibilities in terms of numbers of different alleles, varying dosages of alleles, dominance/co-dominance and linkage phases of the markers. For instance a diploid founder individual has two distinct haplotypes at all loci but the observable genotype at a specific locus may be homozygous if the same observable allele is assigned to both founder haplotypes. While the result of the simulation consists of complete, error-free genotypes it is straightforward to process these to obtain a specified fraction of scoring errors and/or missing data.

## Results and discussion

Our software has a test mode, which allows accumulating statistics of gametes over many meioses in one founder individual. This test mode has been used to validate the simulation results against the theoretical expectations for the simulated models. Here we present results of these simulations.

### Recombination frequency and map length

One series of simulations was performed for an individual with chromosomes of different lengths (50, 100, 200 and 400 cM) with the long arm 4 times as long as the short arm (centromeres at 20% of the chromosome length), in diploids and tetraploids. From each simulated meiosis one randomly selected gamete was sampled; one million meioses were simulated in 100 replications of 10,000 meioses. The allelic constitution of each chromatid was sampled at several loci, spaced such that recombination over intervals of 1, 2, 5, 10, 20, 50, 100, 200 and 400 cM (as far as the chromosome length permitted) at the center and both ends of each chromosome could be studied. The observed recombination fractions were tested against those predicted by the Haldane or Kosambi map functions (depending whether chiasma interference was simulated or not) in the case of bivalents, and against the formulas given in [16] for cross-type and parallel quadrivalents using a t-test, based on the means and standard deviations observed over the 100 replications.

In diploids and in tetraploids with only bivalent formation, with or without chiasma interference, a close agreement was found between the expected and observed amount of recombination; the results for tetraploids are shown in Tables 1a and 2a.

**Table 1:** **Expected and simulated tetrasomic recombination frequencies without chiasma interference**

**a. Two bivalents; expected recombination frequencies according to Haldane map function**													
**Chrom. length**		**50 cM**			**100 cM**			**200 cM**			**400 cM**		
**Interval (cM)**	**Expected recomb**	**Top**	**Center**	**Bottom**	**Top**	**Center**	**Bottom**	**Top**	**Center**	**Bottom**	**Top**	**Center**	**Bottom**
1	0.0099	0.0100	0.0100	0.0098	0.0100	0.0099	0.0099	0.0098	0.0100	0.0099	0.0098	0.0099	0.0099
2	0.0196	0.0196	0.0197	0.0195	0.0197	0.0196	0.0196	0.0196	0.0197	0.0195	0.0196	0.0197	0.0196
5	0.0476	0.0476	0.0478	0.0474	0.0477	0.0475	0.0476	0.0475	0.0479	0.0475	0.0476	0.0476	0.0475
10	0.0906	0.0907	0.0909	0.0905	0.0908	0.0907	0.0907	0.0905	0.0908	0.0906	0.0906	0.0907	0.0908
20	0.1648	0.1648	0.1650	0.1649	0.1650	0.1647	0.1648	0.1647	0.1648	0.1647	0.1647	0.1649	0.1648
50	0.3161		0.3161		0.3161	0.3161	0.3162	0.3160	0.3160	0.3162	0.3161	0.3159	0.3161
100	0.4323					0.4322		0.4322	0.4316	0.4328	0.4317*	0.4322	0.4324
200	0.4908								0.4909		0.4907	0.4910	0.4906
400	0.4998											0.4996	
**b. Cross-type quadrivalents; expected recombination frequencies according to Sved (1964) formula 3**													
**Chrom. length**		**50 cM**			**100 cM**			**200 cM**			**400 cM**		
**Interval (cM)**	**Expected recomb.**^**1**^	**Top**	**Center**	**Bottom**	**Top**	**Center**	**Bottom**	**Top**	**Center**	**Bottom**	**Top**	**Center**	**Bottom**
1	0.0099	0.0095***	0.0096***	0.0095***	0.0096***	0.0096***	0.0095***	0.0099	0.0098	0.0099	0.0099	0.0098	0.0099
2	0.0197	0.0188***	0.0189***	0.0189***	0.0190***	0.0191***	0.0189***	0.0194**	0.0195	0.0194*	0.0195	0.0195	0.0196
5	0.0480	0.0458***	0.0459***	0.0459***	0.0462***	0.0463***	0.0461***	0.0473***	0.0473***	0.0472***	0.0475*	0.0478	0.0477
10	0.0921	0.0878***	0.0879***	0.0878***	0.0886***	0.0891***	0.0884***	0.0902***	0.0907***	0.0904***	0.0907***	0.0916*	0.0910***
20	0.1703	0.1623***	0.1624***	0.1625***	0.1636***	0.1645***	0.1631***	0.1655***	0.1668***	0.1650***	0.1648***	0.1675***	0.1651***
50	0.3420		0.3314***		0.3304***	0.3322***	0.3299***	0.3249***	0.3332***	0.3244***	0.3178***	0.3300***	0.3177***
100	0.5000					0.5054***		0.4834***	0.4971***	0.4833***	0.4446***	0.4824***	0.4442***
200	0.6227								0.6668***		0.5737***	0.6298***	0.5737***
400	0.6874											0.7411***	
**c. Parallel quadrivalents; expected recombination frequencies according to Sved (1964) formula 2**													
**Chrom. length**		**50 cM**			**100 cM**			**200 cM**			**400 cM**		
**Interval (cM)**	**Expected recomb**	**Top**	**Center**	**Bottom**	**Top**	**Center**	**Bottom**	**Top**	**Center**	**Bottom**	**Top**	**Center**	**Bottom**
1	0.0099	0.0099	0.0099	0.0101	0.0100	0.0099	0.0101	0.0100	0.0098	0.0100	0.0099	0.0099	0.0099
2	0.0197	0.0197	0.0197	0.0199*	0.0197	0.0196	0.0198	0.0198	0.0197	0.0197	0.0196	0.0198	0.0197
5	0.0484	0.0482	0.0483	0.0488**	0.0484	0.0482	0.0484	0.0485	0.0483	0.0484	0.0480*	0.0485	0.0484
10	0.0936	0.0934	0.0934	0.0942*	0.0935	0.0933	0.0939	0.0937	0.0936	0.0934	0.0932	0.0940	0.0937
20	0.1756	0.1755	0.1756	0.1760	0.1750	0.1754	0.1760	0.1755	0.1756	0.1755	0.1750	0.1758	0.1757
50	0.3649		0.3654		0.3645	0.3647	0.3652	0.3651	0.3652	0.3649	0.3649	0.3650	0.3651
100	0.5523					0.5520		0.5523	0.5522	0.5526	0.5520	0.5529	0.5523
200	0.6979								0.6982		0.6979	0.6983	0.6978
400	0.7464											0.7467	

*, **, ***: Deviation from expected recombination frequency significant at P < 0.05, P < 0.01 or P < 0.001.

^1^: For cross-type quadrivalents the formula of Sved (1964) assumes a uniform distribution of the chromosome exchange point, whereas our simulations produce a unimodal distribution.

**Table 2:** **Expected and simulated tetrasomic recombination frequencies with chiasma interference according to the Kosambi map function**

**a. Two bivalents; expected recombination frequencies according to Kosambi map function**													
**Chrom. length**		**50 cM**			**100 cM**			**200 cM**			**400 cM**		
**Interval (cM)**	**Expected recomb.**	**Top**	**Center**	**Bottom**	**Top**	**Center**	**Bottom**	**Top**	**Center**	**Bottom**	**Top**	**Center**	**Bottom**
1	0.0100	0.0100	0.0100	0.0100	0.0099	0.0100	0.0099	0.0101	0.0100	0.0100	0.0100	0.0101	0.0100
2	0.0200	0.0199	0.0199	0.0198	0.0200	0.0198*	0.0199	0.0201	0.0200	0.0200	0.0200	0.0201	0.0200
5	0.0498	0.0497	0.0501	0.0496	0.0500	0.0496	0.0497	0.0498	0.0499	0.0500	0.0498	0.0499	0.0500
10	0.0987	0.0988	0.0988	0.0986	0.0993*	0.0989	0.0989	0.0990	0.0991	0.0990	0.0990	0.0988	0.0991
20	0.1900	0.1905*	0.1907**	0.1906*	0.1910**	0.1904	0.1904	0.1907**	0.1907*	0.1906*	0.1908**	0.1906*	0.1909**
50	0.3808		0.3801*		0.3809	0.3807	0.3808	0.3808	0.3806	0.3805	0.3811	0.3803	0.3809
100	0.4820					0.4829*		0.4833**	0.4833***	0.4826	0.4824	0.4825	0.4831**
200	0.4997								0.5000		0.5002	0.5000	0.4995
400	0.5000											0.4997	
**b. Cross-type quadrivalents; expected recombination frequencies derived from Sved (1964) formula 3 applied to diploid recombination according to Kosambi**													
**Chrom. length**		**50 cM**			**100 cM**			**200 cM**			**400 cM**		
**Interval (cM)**	**Expected recomb.**^**1,2**^	**Top**	**Center**	**Bottom**	**Top**	**Center**	**Bottom**	**Top**	**Center**	**Bottom**	**Top**	**Center**	**Bottom**
1	0.0100	0.0094***	0.0091***	0.0092***	0.0096***	0.0093***	0.0096***	0.0100	0.0094***	0.0099	0.0100	0.0095***	0.0099
2	0.0201	0.0186***	0.0182***	0.0184***	0.0191***	0.0185***	0.0193***	0.0199	0.0188***	0.0200	0.0200	0.0192***	0.0199
5	0.0503	0.0460***	0.0453***	0.0460***	0.0480***	0.0461***	0.0479***	0.0494***	0.0470***	0.0496***	0.0497**	0.0478***	0.0496***
10	0.1005	0.0913***	0.0902***	0.0912***	0.0951***	0.0916***	0.0950***	0.0982***	0.0935***	0.0984***	0.0987***	0.0949***	0.0987***
20	0.1975	0.1764***	0.1751***	0.1761***	0.1833***	0.1776***	0.1829***	0.1892***	0.1815***	0.1894***	0.1909***	0.1837***	0.1905***
50	0.4248		0.3736***		0.3794***	0.3782***	0.3790***	0.3825***	0.3800***	0.3824***	0.3806***	0.3798***	0.3813***
100	0.5960					0.5668***		0.5303***	0.5504***	0.5308***	0.4881***	0.5326***	0.4888***
200	0.6815								0.7062***		0.5798***	0.6647***	0.5798***
400	0.7173											0.7480***	
**c. Parallel quadrivalents; expected recombination frequencies derived from Sved (1964) formula 2 applied to diploid recombination according to Kosambi**													
**Chrom. length**		**50 cM**			**100 cM**			**200 cM**			**400 cM**		
**Interval (cM)**	**Expected recomb.**^**2**^	**Top**	**Center**	**Bottom**	**Top**	**Center**	**Bottom**	**Top**	**Center**	**Bottom**	**Top**	**Center**	**Bottom**
1	0.0100	0.0099	0.0101	0.0100	0.0099	0.0100	0.0100	0.0101	0.0101	0.0099	0.0102	0.0101	0.0100
2	0.0201	0.0200	0.0199*	0.0200	0.0200	0.0200	0.0201	0.0201	0.0201	0.0200	0.0201	0.0200	0.0200
5	0.0507	0.0497***	0.0500***	0.0497***	0.0501***	0.0498***	0.0499***	0.0499***	0.0501***	0.0496***	0.0498***	0.0501***	0.0501***
10	0.1023	0.0983***	0.0986***	0.0982***	0.0988***	0.0983***	0.0986***	0.0984***	0.0988***	0.0984***	0.0981***	0.0984***	0.0987***
50	0.4616		0.3918***		0.3923***	0.3921***	0.3920***	0.3918***	0.3921***	0.3914***	0.3916***	0.3915***	0.3925***
100	0.6683					0.5814***		0.5804***	0.5807***	0.5806***	0.5808***	0.5811***	0.5814***
200	0.7443								0.7128***		0.7127***	0.7124***	0.7120***
400	0.7500											0.7479***	

*, **, ***: Deviation from expected recombination frequency significant at P < 0.05, P < 0.01 or P < 0.001.

^1^: For cross-type quadrivalents the formula of Sved (1964) assumes a uniform distribution of the chromosome exchange point, whereas our simulations produce a unimodal distribution.

^2^: The formulas of Sved (1964) assume no chiasma interference. They express the tetrasomic recombination frequencies as a function of the corresponding disomic recombination on the same interval. Here we use the disomic recombination according to Kosambi map function as input in these formula's.

In tetraploids with only cross-type or parallel quadrivalent formation the amount of recombination increased to values above 50%, as expected [16]. For these configurations the Haldane and Kosambi map functions are not valid. However, for the situation without chiasma interference, [16] has given formulas for the cross-type and parallel quadrivalent configurations. The formula for cross-type quadrivalents however was based on an assumed uniform distribution of the chromosome exchange point, whereas in our model this has a unimodal distribution with a maximum at the chromosome center. This could explain why our results deviate significantly from the formula of [16] for cross-type quadrivalents (Table 1b). As far as we are aware there is no experimental evidence available for either a uniform or a unimodal distribution of the exchange point. Still, the simulated level of recombination in cross-type quadrivalents is in between that of bivalents and parallel quadrivalents, in accordance with [16]. For parallel quadrivalents our simulation results agree perfectly with [16] (Table 1c).

We also present the corresponding simulated values for cross-type and parallel quadrivalents with chiasma interference (Table 2b and c). For those situations no theoretical expectations are available. We have attempted to apply the formulas in [16] to the situation with chiasma interference by applying them to the recombination fractions derived from Kosambi’s instead of Haldane’s map function, but the simulated results deviate significantly from the values predicted by this method.

### Double reduction

**Figure 2:**
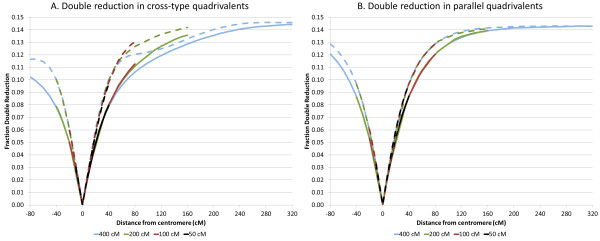
**Double reduction frequencies of (A) cross-type and (B) parallel quadrivalents of chromosomes of 50, 100, 200 and 400 cM with the centromere at 20% of the length.** Solid and dashed lines are derived from simulations without or with chiasma interference respectively.

In the simulations discussed above we also calculated the frequency of double reduction (DR) at various positions on the chromosomes. As expected, for the configuration without quadrivalents no DR was observed at any position. With both cross-type and parallel quadrivalents the frequency of DR increased from 0 at the centromere to a maximum of 1/7 at large distances from the centromere, in cross-type and parallel quadrivalents (Figures 2A and B). These simulations were done with a random partitioning of the centromeres in the first meiotic division, meaning that for a given pair of recombined chromatids there is a probability of 1/3 that they end up at the same pole. The observed limit of 1/7 corresponds to the theoretical probability that a given allele will meet its matching allele in a gamete when the alleles are distributed randomly, since of the alleles on the other 7 chromatids only one is on its sister chromatid. A random assortment of alleles is what would be expected for loci very distant from the centromere. The value observed in our simulations is in the range of maximum DR frequencies mentioned in the literature based on varying theoretical considerations. For instance [17] assumes that the maximum fraction is 1/8, while a maximum value of 1/6 is also mentioned [18,23] and [24] even mentions 1/4.

For parallel quadrivalents the frequency of DR at a given distance from the centromere is not dependent on the length of the chromosome. In cross-type quadrivalents however, starting at a distance of about 50 cM from the centromere the amount of DR is somewhat smaller in longer chromosomes. In the longest simulated chromosome (400 cM) with chiasma interference the slope of the curve does not decrease monotonically; around the center of the chromosome the amount of DR is less than might be expected (Figure 2A). We have no explanation for this, but noted that the distribution of the chromosome exchange point has also a maximum at the chromosome center that is more pronounced in longer chromosomes; the two phenomena might be related.

### Quadrivalent vs. bivalents formation

The results above were obtained with 100% bivalent or 100% quadrivalent formation. We also simulated meioses with “natural pairing” and preferential pairing varying from 0% (completely random pairing), 25%, 50%, 75% to 100% (only pairing between matching pairs). With a given probability of preferential pairing p the probability of pairing between the two homologues is p + (1-p)/3, and the other two possible pairings have probability (1-p)/3. Two bivalents result when the pairing at both ends is identical, so the expected fraction of meioses with two bivalents P_bivalents_ = (p + (1-p)/3)^2^ + 2((1-p/3)^2^, and the fraction meioses with a quadrivalents is 1-P_bivalents_ (equivalent formulas with a different parameterization are presented in [4]). For completely random pairing (p = 0) the expected fraction of quadrivalents is therefore 2/3, in agreement with [14]. The expected and simulated frequencies of quadrivalents are in close agreement (Table 3).

**Table 3:** **Expected and simulated frequencies of quadrivalent formation**

P (preferential pairing)^1^	0	0.25	0.5	0.75	1
expected freq. quadrivalents	0.667	0.625	0.500	0.292	0.000
simulated freq. quadrivalents^2^	666828	625628	500039	291589	0

^1^ The probability that chromosome pairing at the telomeres is preferential (as opposed to random).

^2^ Results of a simulation of 1,000,000 meioses.

### Generation of phenotypes

After the simulated genotypes have been obtained, a possible next step is the generation of phenotypes, given the simulated allelic composition of the individuals. This is not implemented in the simulation program, but can be done for any genotype-to-phenotype model, including simple dominant or intermediate qualitative traits but also quantitative traits affected by environmental variation as well as genetic effects. Also epistatic interactions can be modelled in this way. Dosage effects of alleles on the phenotype or effects of interactions between alleles within or between loci in a tetraploid can therefore also be included easily.

## Conclusion

The software package PedigreeSim allows to simulate diploid and tetraploid populations according to various genetic models. The simulation results obtained with models for which theoretical expectations are available closely match these expectations. As the implementation of other models, especially the models involving quadrivalent formation in presence of chiasma interference, is a combination of elements that separately have been shown to work as expected (i.e. chiasma interference in bivalents, and quadrivalents without chiasma interference) it may be assumed that the results obtained for these models are reliable as well.

In principle it is relatively straightforward to extend the approach presented here to hexaploids and higher ploidy levels. In these cases meioses will generally consist of combinations of bivalents, quadrivalents and possibly structures consisting of six or more chromosomes, where only for those complex structures new code would be needed. However there is little theoretical and experimental information available on the recombination and segregation in higher (auto)polyploids, so the validity of such extensions would not be clear.

This software is particularly relevant for genetic analysis of allotetraploid and autotetraploid crops as it allows generation of populations of any size and under different inheritance models in tetraploids such as disomic inheritance, tetrasomic inheritance with random pairs of bivalents or with quadrivalent formation. PedigreeSim will allow comparison of observations from experimental crosses against these possible inheritance models. It will also allow studies of the effectiveness of different steps in the construction of maps and haplotypes and in QTL analysis of polyploids. We expect therefore that PedigreeSim will further the development of methods for genetic analysis in tetraploids.

### Availability and requirements

Project name: PedigreeSim

Project home page: The current version of the program and manual are available without cost from http://www.plantbreeding.nl/UK/software_pedigreeSim.html

Programming language: Java

Operating systems: PedigreeSim can be used on any platform for which a Java Virtual Machine is available, which includes all versions of MS Windows, Linux and Apple operating systems.

Availability: The program source code, the compiled version, the manual and example files are available from the project home page and as Additional files 1, 2, 3, and 4 with this article.

The source consists of classes representing biological entities (Individual, Gamete, Chromosome, Bivalent, Quadrivalent etc.) with biological functions (generateChiasma, doMeiosis, mating etc.). Also a considerable amount of explanation is included in the form of comments. This makes the code relatively easy to understand for biologists and allows to build upon this code to enhance functionality, extend it to higher ploidy levels etc.

Licensing: PedigreeSim itself is distributed under the GNU General Public License (GPL) version 2 or later ( http://www.gnu.org). PedigreeSim makes use of the jsci-core library of JScience, which is enclosed in the file with the compiled version along with a text document detailing the conditions under which it may be used and distributed.

Compatibility with other software: Input and output files are plain tab-delimited text files, easy to compose manually and to import and export to and from other software. Their layout is discussed in the manual, which is included as Additional files 1, 2, 3 and 4 and which is also available from the project homepage.

## Competing interests

The authors declare that they have no competing interests.

## Author’s contributions

REV wrote the PedigreeSim software, carried out the simulations and drafted the manuscript. CAM initiated the research project and helped to draft the manuscript. Both authors read and approved the final manuscript.

## Supplementary Material

Additional file 1 Archive containing the compiled version of PedigreeSim and instructions on how to run it. Click here for file

Additional file 2 Archive containing the full source code of PedigreeSim. Click here for file

Additional file 3 The PedigreeSim Manual. Click here for file

Additional file 4: Archive containing the example input files for PedigreeSim. Click here for file
