# Supplementary material for: The simulation of meiosis in diploid and tetraploid organisms using various genetic models
Source: BMC Bioinformatics. 2012 Sep 26;13:248. doi: 10.1186/1471-2105-13-248 (PMC3542247; doi:10.1186/1471-2105-13-248)
Supplement: Additional file 3 — The PedigreeSim Manual. [file 1471-2105-13-248-S3.pdf]

PedigreeSim Manual

Simulation of genotypes in pedigrees of diploid and tetraploid species

Roeland E. Voorrips, 2012
Wageningen University and Research Center, Department of Plant Breeding.
P.O. Box 16, 6700 AA Wageningen, the Netherlands
e-mail: roeland.voorrips@wur.nl

Contents

Introduction 2
Citation 2
Description of the simulation process 2
Operation 3
  Parameter file 3
Input files 5
  Pedigree file 5
  Chromosome file 5
  Linkage map file 5
  Founder genotypes file 6
  Haplostruct files 7
Output files 7
  Pedigree file 7
  Haplostruct files 7
  Genotypes file 8
  Founderalleles file 8
  Alleledose file 8
Simulation of missing data, scoring errors and phenotypes 8
Test mode 8

Introduction

PedigreeSim is software that generates simulated genetic marker data of individuals in pedigreed populations. A population can consist of either diploid or tetraploid individuals. PedigreeSim can simulate meioses with or without chiasma interference, and in tetraploids it can simulate meioses with two bivalents or one quadrivalent per set of hom(oe)ologous chromosomes. Also varying probabilities of preferential versus random pairing of chromosomes can be simulated, allowing to simulate amphidiploids as well as autotetraploids and intermediate forms.

Input and output files are text files in simple tabular form that are easily produced or read by other software including Excel, R and other statistical packages.

PedigreeSim is written in Java, which means that it is available on any platform where Java is available, including among others all distributions and versions of Linux, Windows and Apple OS.

Citation

Voorrips RE, Maliepaard CA (2012) The simulation of meiosis in diploid and tetraploid organisms using various genetic models. BMC BioInformatics (submitted)

Description of the simulation process

The simulation is based on two types of data: the genome structure of the species and the pedigree. The genome structure is specified by the ploidy, the absence or occurrence of chiasma interference, and by the properties of the chromosomes, which include their length and centromere positions, and in the case of tetraploids also their probabilities of preferential pairing (versus random pairing) and of quadrivalent formation (versus formation of two random bivalents). The pedigree is a list of all individuals specifying their parents. Founders of the population have no parents; all individuals must have either two known parents (which may be identical in the case of selfing) or no known parents (in the case of founders); it is not allowed to specify one parent as known and the other as unknown.

Each of the founders is assigned its own "founder alleles" over the whole length of all chromosomes. For instance, in a diploid the first founder has two founder alleles 0 and 1, the second founder has alleles 2 and 3 and so on, for every position on all chromosomes. In a tetraploid this would be alleles 0, 1, 2, 3 for the first founder, alleles 4, 5, 6, 7 for the second founder and so on.

Then gametes are produced and combined according to the pedigree and taking into account the probabilities of different chromosomal pairing modes and meiotic configurations as listed above. A chromosome in a gamete or a non-founder individual consists of segments of different founder alleles, with recombinations at specific positions distributed according to models without or with chiasma interference. Such a pattern of segments of different founder alleles we call a "haplostruct".

In this way the haplostructs (the patterns of founder allele segments) for all chromosomes of all individuals in the pedigree are obtained. The final stage involves the assignment of observed marker alleles to each founder allele. This is performed by specifying a linkage map with marker loci, and ordered genotypes for the founders at the locus positions on the map. For each locus on the map the observed marker genotype of an individual is determined by checking the haplostructs to find the founder alleles at the locus position and looking up the corresponding marker allele at that position.

The final step can be repeated with another map or other founder genotypes, using the same simulated haplostructs.

Operation

One way of starting PedigreeSim is by typing the following line in the console:

java -jar PedigreeSim.jar

(if PedigreeSim.jar is not in the current directory you add the path)

When started this way there should be a parameter file with the name PedigreeSim.par in the current directory. Alternatively you can specify a parameter file:

java -jar PedigreeSim.jar example.par

(again with path for PedigreeSim.jar and example.par if needed)

Parameter file

The parameter file specifies the input and output files, and a few other parameters. A typical example is shown below:

PLOIDY = 4
MAPFUNCTION = HALDANE
MISSING = NA
CHROMFILE = example.chrom
PEDFILE = example.ped
MAPFILE = example.map
FOUNDERFILE = example.gen
OUTPUT = example_out

The first two lines specify the ploidy (currently only diploid (2) and tetraploid (4) are supported), and the map function: Haldane (no chiasma interference modelled; in bivalents recombination is in accordance with the Haldane map function) of Kosambi (with chiasma interference modelled such that for bivalents the recombination is in accordance with the Kosambi map function). The third line specifies the string used to indicate a missing value; if this is absent "NA" is assumed for compatibility with R, so in the example this line could have been omitted. Note that the same symbol for missing data is used for all input and output files. Names or identifiers for individuals, chromosomes, loci and marker alleles cannot be the same as this "missing" symbol.

The next four lines specify input data files; these are discussed in the next sections. The last line is the basic filename of the output files; to this string further parts are appended for the different files.

All keywords in the parameter file may be entered in upper and/or lowercase; spaces are allowed but not required around the "=" sign. The case is relevant for the "missing" string, and for filenames if the operating system is sensitive to the case. Blank lines and comment lines (starting with a semicolon) may be added, as well as extra text after the data on a line. The only requirement is that all the data (filenames and other strings) do not contain blanks (spaces, tab characters).

Instead of supplying a pedigree file a standard population type and size may be specified. For example, by removing the line

PEDFILE = example.ped

and adding the lines:

POPTYPE = F1
POPSIZE = 150

a pedigree will be generated with two parents (founders) named P1 and P2 and 150 F1 individuals. There are four standard population types: S1 (selfings of one parent), F1 (full-sib progeny of two

parents), F2 (two parents, one F1 individual and an F2 consisting of selfings of the F1) and BC (two parents, an F1 and a progeny obtained by backcrossing the F1 to the first parent). The POPSIZE keyword refers to the size of the progeny, excluding parent(s) and possible intermediate F1 individual. Any other type of population can be simulated by specifying it in a pedigree file.

The lines specifying the map file and the founder genotypes may be omitted; in that case only the files with the simulated haplostructs (example_out.hsa and example_out.hsb) are produced. These can be used in later runs to generate genotypes of the full population based on different map and founder genotype files:

A line specifying haplostructs from an earlier simulation for the same population may be specified:

HAPLOSTRUCT = myhaplostruct

In that case no new simulation of the pedigree is performed, only the marker genotypes of all individuals are calculated based on the supplied haplostruct files myhaplostruct.hsa and myhaplostruct.hsb, the linkage map and the marker genotypes of the founders.

There are several other parameters that affect the way in which gamete formation proceeds. They all have a default value as specified below and therefore can be omitted from the parameter file:

ALLOWNOCHIASMATA = 0 or 1 (default 1): if 1, meiosis is taken to proceed normally even if some chromosomes do not have any chiasmata; this results in the expected amount of recombination according to the Haldane or Kosambi map functions (in other words, the chosen map function applies).
If ALLOWNOCHIASMATA=0, the chiasma generation process is repeated for each Bivalent or Quadrivalent until at least one chromatid of each parental chromosome is involved in at least one chiasma. This option illustrates the consequences of the idea that chromosomes without chiasmata tend to be lost later in meiosis, leading to non-functional gametes. In this situation the mean interval length between chiasmata is adapted such that on average one chiasma per chromatid per Morgan is generated, which means that the mean distance between chiasmata depends on the chromosome length. In bivalents the recombination between the chromosome ends will always be 0.5 and hence the effective genetic chromosome length will be infinite. A minimum chromosome length of 70 cM is required in this case; chromosomes of 50 cM or shorter are by definition impossible.

NATURALPAIRING = 0 or 1 (default 1): if 1, the configuration in the tetraploid meiosis (i.e. one quadrivalents or two bivalents) is determined by the pairing at the chromosome ends. In absence of preferential pairing this leads to 2/3 quadrivalent and 1/3 bivalent configurations; with 100% preferential pairing only bivalents are generated. If this parameter is 0, the fractions of bivalent vs quadrivalent configurations are determined by the “quadrivalents” parameter in the Chromosome file.

PARALLELQUADRIVALENTS = 0.0 – 1.0 (default 0.0): in tetraploids, the 4 chromosomes can either form 2 bivalents or one quadrivalent (the probability of both is determined by the NATURALPAIRING parameter (above) and the prefPairing and Quadrivalents parameter in the Chromosome file). Quadrivalents are most realistically modelled as a cross-like structure; in each of the four branches two chromosomes are paired and where the branches meet the chromosomes change partners. A less realistic model, which is also sometimes mentioned in literature, is that where all four chromosomes are aligned in parallel over their full lengths and any chiasma can involve any pair of chromosomes. This parameter specifies the probability of the latter configuration (range 0.0-1.0); the default is 0.0, i.e. all quadrivalents are in the cross configuration.

PAIREDCENTROMERES = 0.0 – 1.0 (default 0.0): in a cross-type quadrivalent, two models are suggested in literature about the way that the four centromeres (and the attached, possibly recombined chromatids) are distributed over the two poles in the first meiotic division. One model is that the four centromeres can be divided into two pairs in any of the three possible ways (ab/cd, ac/bd, ad/bc) with equal probability; the second model assumes that the centromeres are paired according to the quadrivalent branch in which they end up, and that from each of the two pairs one centromere moves to either pole. This parameter specifies the probability of the second model (range 0.0-1.0); the default is 0.0, i.e. all cross-type quadrivalents segregate according to the first model.

Input files

Pedigree file

The pedigree file is a file containing a list of all individuals and their parents. An example (example.ped) follows:

Name	Parent1	Parent2
P1	NA	NA
P2	NA	NA
F1_1	P1	P2
F1_2	P1	P2
F1_3	P1	P2
F1_4	P1	P2
F1_5	P1	P2

The header line must be present (upper/lower case not relevant). The next lines each specify one individual. For each individual either both parents must be missing (for founders, as in the first two individuals) or both parents must be known (they may be identical, to specify a selfing). The string used to indicate a missing parent must be specified by the MISSING parameter in the parameter file, if different from the default. All names must be separated by blanks (tabs and/or spaces) and names may not contain blanks. Empty lines and lines starting with a semicolon are allowed and ignored, which allows to "comment out" some individuals.

The pedigree does not have to be sorted; PedigreeSim will sort the pedigree such that each individual appears after both parents. If this is already the case no sorting will be done. If sorting is done, or if the pedigree is generated automatically by specifying a standard population type a file <output>.ped is written containing the sorted pedigree.

Chromosome file

The chromosome file is a file containing the properties of all chromosomes. An example (example.chrom) is as follows:

chromosome	length	centromere	prefPairing	quadrivalents
A	100.0	20.0	1.00	0.0
B	100.0	20.0	0.67	1.0

For diploid populations the last two columns may be omitted. The header line (at least the first 3 captions, upper/lower case not relevant) is required. The subsequent lines each list one chromosome, with the name, the length and centromere positions (in cM) and two probabilities that are only relevant in the case of tetraploids (see below). The chromosome name must not contain spaces. The columns must be separated by blanks (spaces or tabs) and all except the first may only contain numbers. No missing data are allowed. Empty lines and lines starting with a semicolon are allowed and are ignored, which allows to "comment out" some chromosomes.
The prefPairing column specifies the amount of preferential paining in tetraploids, from 0.0 (all combinations have equal probability, as in autotetraploids) to 1.0 (fully preferential pairing, as in amphidiploids).
The quadrivalents column specifies the fraction of quadrivalents vs bivalents. This column has no effect (but must still be present) when in the parameter file NATURALPAIRING = 1 (the default value): in that case the fraction quadrivalents arises automatically from the pairing process at the telomeres.

Linkage map file

The linkage map consists of a list of locus names with the chromosome and the positions (in cM) on the chromosomes. For example (example.map):

marker	chromosome	position
A000	A	0
A025	A	25
A050	A	50
A100	A	100
B000	B	0
B025	B	25
B050	B	50
B100	B	100

The header line must be present (upper/lower case not relevant). The next lines must list the loci in map order, with the chromosomes in the same order as in the chromosome file. The locus positions must fit within the chromosome lengths specified in the chromosome file (but they do not need to cover the full length of the chromosome). At least one locus must be specified for each chromosome, and the chromosome names must match exactly (upper/lower case relevant) with those in the chromosomes file. The columns must be separated by blanks (tabs and/or spaces) and names may not contain blanks. No missing data are allowed. Empty lines and lines starting with a semicolon are allowed and are ignored, which allows to "comment out" some loci.

Founder genotypes file

In this file the actual genotypes of the founder individuals for all loci in the map are specified. Therefore, if a founder genotypes file is given, also a map file (see previous section) must be given.

The marker genotypes are ordered: for each founder there are two (diploids) or four (tetraploids) columns. The marker alleles in one column all belong to the same founder allele; for loci on the same chromosome this means that they are in coupling phase. An example of a founder genotypes file for a tetraploid population with two founders and the linkage map given in the previous section might look as follows (example.gen):

marker	P1_1	P1_2	P1_3	P1_4	P2_1	P2_2	P2_3	P2_4
A000	q	q	Q	q	Q	Q	Q	Q
A025	A	T	A	T	A	T	A	T
A050	210	214	216	214	214	214	214	214
A100	312	312	312	312	310	314	312	314
B000	one	two	three	four	five	six	seven	eight
B025	G	A	G	G	G	G	G	G
B050	1	0	1	0	0	0	0	0
B100	x	y	y	y	z	z	z	z

The header line must start with "marker". After this follow two or four column captions per founder, each consisting of the name of the founder (upper/lowercase relevant), an underscore and a number (1-2 or 1-4). The order of the founders is not relevant, but the order of the columns within each founder is.

Below that each line specifies the genotypes for one marker. The markers must be given in the order of the linkage map (even if two markers are at the same position their order must be as given in the map file) and all markers on the map must be present. After the marker name (uppercase/lowercase identical to the name in the linkage map) a marker allele is specified for each column of each founder. Note that any string of letters, digits and punctuation is allowed in a marker allele except blanks (spaces or tabs) as these are used to separate the columns. This allows for example to represent dominant/recessive alleles (Q/q), SNP markers (A/T etc.), multi-allelic SSR markers (210/214/216) and any other type of QTL, major gene or marker alleles. Names of founders (in the column captions) and loci (first column) must match exactly (upper/lower case relevant) with those in the pedigree file

and map file. The parents of standard populations have the names P1 and P2, or only P1 in the case of an S1 (selfing) population. No missing data are allowed. Empty lines and lines starting with a semicolon are allowed and are ignored.

It is important to understand the difference between the “founder allele” and the “actual allele” at a given locus. Founder individuals have two (diploids) or four (tetraploids) different founder alleles at each locus; if the same founder allele is present at that locus in another individual in the pedigree it is identical-by-descent (IBD) to the allele in that founder. Different founders do not share the same founder alleles. In contrast, “actual alleles” are the observed alleles. A SNP locus for instance has only two observable alleles. A diploid founder that is homozygous for the SNP locus has two different founder alleles, but two times the same actual allele at that locus. The Founder genotypes file specifies the link between the observed, actual alleles and the IBD founder alleles. See also the section Description of the simulation process.

Haplostruct files

If the parameter file specifies the HAPLOSTRUCT keyword, a pair of Haplostruct files with extensions .hsa and .hsb must be present. These files are produced by PedigreeSim itself and are discussed under Output files.

Output files

All filenames of output files start with the string specified by parameter OUTPUT in the parameter file; in the following sections this string is represented as <output>. The files are created in the current working directory and will overwrite existing files with the same name. Depending on the contents of the parameter file some of the following output files are generated:

Pedigree file

If a standard population is specified or if a pedigree file required sorting, the generated or sorted pedigree is written to <output>.ped. The format is described as above; no empty lines or comments are present and columns are separated by tabs.

Haplostruct files

Unless the haplostruct files are specified in the parameter file (in which case they are used as input files) the simulated haplostructs are written to a pair of tab-separated files <output>.hsa and <output>.hsb. Together these files specify how the chromosomes of all individuals are composed from segments of the founder chromosomes:

File <output>.hsa specifies on each line first (1) the individual), (2) the chromosome and (3) the homolog number (1-2 for diploids, 1-4 for tetraploids). For the chromosome identified in this way then follows a series of numbers: these identify the founder alleles that make up the segments of the chromosome. The number of columns in the file is fitted to the chromosome in the population with the largest number of founder segments. For all chromosomes with fewer segments the remaining columns are filled with the “missing” symbol. Every line therefore has the same number of items, which facilitates reading by other software. Also, as each chromosome is composed of at least one founder segment, there is at least one non-missing number after the first three columns in each line.

File <output>.hsb specifies the positions (in cM) of the recombination points between the segments. The lines do not include the individual, chromosome and homolog number but are in the same order as in file <output>.hsa. Like in that file, all lines have the same number of items, equal to the maximum number of recombination points in any chromosome in the population. The lines are filled out with the “missing” symbol for chromosomes with less recombination points. Chromosomes consisting of only one segment (as in the founders) have no recombination points and therefore have lines with only missing values.

Genotypes file

The Genotypes file lists the observed marker genotypes for all individuals and all loci. The format of the file and of the column captions is exactly as described for the Founder genotypes file above. The filename of the genotypes file is <output>_genotypes.dat. It is generated only if a map file and a founder genotypes file are specified in the parameter file.

Founderalleles file

The Founderalleles file (<output>_founderalleles.dat) is very similar to the Genotypes file (see previous section). In this file for all loci and all individuals the founder alleles present at the locus positions are given, instead of the observed marker alleles individuals (for the distinction between founder alleles and observed or actual alleles see section Founder genotypes file). The Founderalleles file is generated if a map file is specified.

Alleledose file

The Alleledose file (<output>_alleledose.dat) gives the allele dosage for all individuals, for the (alphabetically) highest marker allele at each locus. The allele dosage varies 0-2 in diploids and 0-4 in tetraploids. The layout of the Alleledose file is similar to that of the Genotypes and Founderalleles files, but here only one column per individual is present and the caption of that column is the individual's name.
If no founder genotype file is given, the dosage of the lowest founder allele (the first allele of the first founder) is given.

Simulation of missing data, scoring errors and phenotypes

PedigreeSim generates complete, error-free genotypic data. It is straightforward to introduce scoring errors and missing values at some or all loci by importing the Genotypes file into Excel, R or other software and manipulating the data.
Similarly it is possible to calculate phenotypic trait values from the genotypic data at one or more loci, using any genotype-to-phenotype model. This may be a simple one-locus qualitative model or a complex quantitative model involving epistasis among multiple loci and environmental variation. Obviously these phenotypic data should be calculated from the complete, error-free genotypic data.

Test mode

Apart from the simulation of genotypes in pedigrees, PedigreeSim can also be used to study the properties of the gamete generation process. The most flexible way is to use the provided Java classes and their methods to write a dedicated program for studying or testing a specific feature. But PedigreeSim also has a built-in possibility to study at least some aspects.

By adding the line
TEST = 1
to the parameter file, PedigreeSim will not produce the normal files with pedigree genotypes, but instead it will perform a series of meioses from one fully heterozygous parent. The number of meiosis per iteration is defined by the POPSIZE keyword. The new keyword TESTITER defines the number of iterations. Of the other parameters, PLOIDY, MAPFUNCTION, CHROMFILE, MAPFILE and OUTPUT are still needed and have the same meaning as before.
This will produce a file <output>.dat, which for each chromosome lists a number of statistics based on sampling one gamete per meiosis, including the recombination frequencies between all pairs of markers, a test of the significance of the differences between the observed recombination frequencies and those expected according to the Haldane or Kosambi map (if more than 2 iterations are performed), the frequencies of the different genotypes at each locus, a frequency distribution of the number of recombination points and of the number of different founder alleles per chromosome. If PLOIDY=4 also the frequency of homozygosity (due to double reduction) of the gametes at all markers, and a frequency distribution of the position of the chromosome exchange points in cross-type quadrivalents is listed. Several additional parameters are provided to specify which output is needed:
PrintGametes = 0 (default), 1 or 2. If 0, no gametes are printed; if 1, the one sampled gamete per meiosis is printed, and if 2, all gametes are printed. Each gamete contains (ploidy/2) haplostructs per chromosome, each of which is printed on a separate line. This option can therefore lead to a large

amount of output. A haplostruct is printed as a sequence of integers (the founder alleles of the successive segments) alternated with real numbers (the recombination positions).
PrintEachIter = 0 (default) or 1. If 1, for every iteration statistics are printed based on all generated alleles and on the one selected gamete per meiosis. If the number of iterations is large this option again can give a large amount of output.
PrintMapdistances = 0 (default) or 1. If 1, in addition to the recombination frequencies between all pairs of loci, also the corresponding map distances (based on the Haldane or Kosambi map functions) are printed, and tested against the given map distances. This test is only valid for bivalents, therefore this option is off by default.

One further keyword exists, which would normally only be used for test purposes:

BIVALENTSBIDIRECTIONAL = 0 (default): usually in a Bivalent we generate the chiasmata sequentially, moving from the start to the end of the chromosome. In a cross-type quadrivalent however we generate the chiasmata starting from the ends of the four arms, and a solution is needed (especially in the case of chiasma interference / the Kosambi map function) to decide whether a new chiasma still fits between the previous chiasma on the same arm and the chiasmata already present on the other arms. By setting this parameter to 1 (or true) we apply the same method to bivalents: chiasmata are generated alternating from the start and the end of the chromosome, and a decision is made whether each new chiasma still fits. A comparison of the recombination frequencies and inferred map lengths with this parameter set to 0 or 1 has shown that no difference is detectable in the results, meaning that the procedure used in quadrivalents is valid.
